# Gender differences in the nonmedical use of psychoactive medications in the school population- national trends and related factors

**DOI:** 10.1186/s12887-019-1728-8

**Published:** 2019-10-21

**Authors:** Pilar Carrasco-Garrido, Isabel Jiménez-Trujillo, Valentín Hernández-Barrera, Nazaret Alonso-Fernández, Soledad García-Gómez-Heras, Domingo Palacios-Ceña

**Affiliations:** 1 Preventive Medicine and Public Health Teaching and Research Unit, Health Sciences Faculty, Universidad Rey Juan Carlos, Av. Atenas s/n. 28 922, Alcorcon, Madrid Spain; 2 Department of Basic Health Sciences, Health Sciences Faculty, Universidad Rey Juan Carlos, Av. Atenas s/n. 28 922, Alcorcon, Madrid Spain; 3 Department of Physical Therapy, Occupational Therapy, Rehabilitation and Physical Medicine, Universidad Rey Juan Carlos, Av. Atenas s/n. 28 922, Alcorcon, Madrid Spain

**Keywords:** High-school students, Nonmedical use of prescription drugs, Gender, National Survey on drug use

## Abstract

**Background:**

The nonmedical use of prescribed medicines among adolescents has increased significantly in recent years. Our study was designed to describe the prevalence of the nonmedical use of tranquilizers, sedatives, and sleeping pills (TSSp) among the school-age population residing in Spain from a gender perspective, and to identify factors associated with such use.

**Methods:**

Nationwide, epidemiological, cross-sectional study on the nonmedical use during the previous 30 days, of TSSp by the Spanish school population. We used individualized secondary data retrieved from the 2004, 2006, 2008, 2010, 2012 and 2014 Spanish state survey on Drug Use in Secondary Education and a total of 179,114 surveys from respondents aged 14 to 18 years. Using logistic multivariate regression models, we estimated the independent effect of each of these variables on the nonmedical use of medicines. Two models were generated- one for females and one for males.

**Results:**

2.86% (5116) of the Spanish school population of both sexes made nonmedical use of TSSp. Prevalence was greater among girls than among boys for all the study years. Patterns of nonmedical use among female adolescents were related to alcohol, tobacco and marijuana use. Consumption of illegal psychoactive substances, other than marijuana, was the variable showing the greatest value among male teenagers (aOR 6.21 (95% CI 4.97–7.77).

**Conclusions:**

The prevalence of the nonmedical use of TSSp is higher in girls than in boys. The influence of legal and illegal psychoactive substances leads to a higher likelihood of nonmedical use of TSSp in high-school students in Spain.

## Background

The latest World Drug Report [1] indicates that the period from early adolescence (12–14 years of age) to late adolescence (15–17) is one of high risk for starting psychoactive substance consumption. Research shows that the nonmedical use of prescribed medicines among adolescents has increased significantly in recent years [2, 3] reaching values close to 5% among US teenagers [4] and 9% among European teens [5]. Consumption prevalence for prescription medications such as psychodrugs has increased [6], but an increase in the nonmedical use of these drugs has also been observed among these teenagers [7]. This situation is reflected in the results of the National Survey on Drug Use and Health (NSDUH), and the European School Survey Project on Alcohol and other Drugs (ESPAD), with respect to the nonmedical use of tranquilizers and sedatives among the adolescent population of these countries [8, 9].

Gender differences in psychoactive substance use rates have been consistently studied among the general population. Men present the significantly highest rates of consumption, abuse and dependence values [10–12], although women present certain specific patterns of substance use [13–15].

The influence of gender on drug consumption habits is conditioned by a generational factor. For the adult population, who were mostly brought up with traditional gender role models, consumption among women is much lower than among men. On the other hand, for current adolescents, who are experiencing more egalitarian gender role models, a trend towards parity of drug consumption habits can be observed with parity already reached for legal psychoactive substances, such as tobacco and alcohol, where the lessening of the consumption gap by female teenagers is evident [16, 17].

Psychotropic drugs represent a type of legal psychoactive substance that affects all age groups of men and women differently with regard to gender for both medically prescribed drugs and drugs ingested for nonmedical use [18–20].

The scientific literature shows that the psychoactive substances that women consume tend to be legal psychoactive substances that are socially acceptable. This makes their consumption more likely to go unnoticed, becoming practically invisible to those who work in the drug additions field and who, as a result, focus more frequently on consumption by the male population. This situation is especially relevant for adolescent females, whose consumption of legal drugs is clearly increasing and has surpassed males consumption of psychoactive substances including alcohol, tranquilizers, and tobacco [21, 22].

Studies have been published that analyzed factors that may influence the nonmedical use of psychodrugs among adolescent populations, and predicting factors identified for the nonmedical use of medications include age, gender, relationship with parents, school absenteeism, family environment and friends [23, 24], and the consumption of other psychoactive drugs [7]. However, there are too few studies specifically analyzing the differences between males and females regarding the nonmedical use of these psychoactive substances with respect to sociodemographic factors, substance abuse, and other variables that could act as predicting factors.

The objective of our study was to examine, from a gender perspective, the nonmedical use prevalence of tranquilizers, sedatives, and sleeping pills (TSSp) among the Spanish population and to identify factors associated with such consumption. We also assessed the evolution of nonmedical use of these drugs from 2004 to 2014 among the adolescent population of both sexes.

## Methods

An analysis was performed on the data collected in the Spanish State Survey on Drug Use in Secondary Education (ESTUDES), as well as on the data from the six surveys conducted between 2004 and 2014. A descriptive, cross-sectional study was conducted on the nonmedical use of TSSp by Spanish adolescents. School population was the population of our study, based on data from 11,716 adolescents of both sexes aged 14 to 18 years. Details of the Spanish State Survey on Drug Use in Secondary Education methodology have been previously published in a related article [25].

As dependent dichotomous variables, “yes” or “no” answers were taken to the question, Have you taken a tranquilizer, sedative, and/or sleeping pill without a prescription during the last month?

As independent variables, we firstly analyzed a number of sociodemographic characteristics and then we continued with variables related to the use of other legal and illegal psychoactive drugs. For the analysis, we used certain variables related to the adolescent population context, such as risk perceived for consumption, ease of obtaining psychodrugs or information previously received on drugs.

Descriptive statistics of the main study variables have been used to calculate the nonmedical use of TSSp prevalence during the years 2006 to 2014. All calculations were used for both sexes. A generalized linear model with a binomial response and logit link function (logistic regression), using the year as the continuous variable was employed for the bivariate analysis of the changes in the variables by year.

The six EDADES surveys were grouped in order to be analyzed globally, and thus increase the power of contrasts.

The raw odds ratio (OR) and its 95% confidence interval (95% CI) were used so as to determine the association between the non-medical use of TSSp and the study variables. Subsequently, two multivariate analysis models were designed (one for males and the other for females) using logistic regression models, thus obtaining the corresponding adjusted OR and 95% CI. Finally, trends of nonmedical use of TSSp from 2004 to 2014 were analyzed both in males and in females, with their corresponding odds ratios.

The svy function (survey command) of the STATA program (STATA Corp, College Station, Texas, USA) was used in the estimates, with the incorporation of the sampling design and weights of the statistical calculations already specifically developed in the study. Statistical significance was considered as a 2-tailed α < 0.05.

## Results

**Fig. 1:**
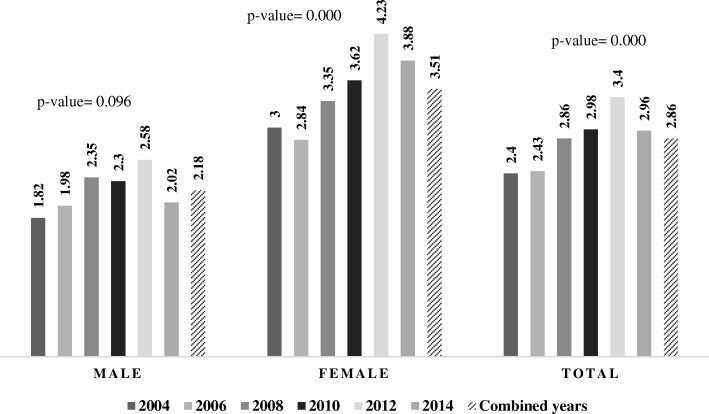
Prevalence (%) of nonmedical use of tranquilizers, sedatives, and sleeping pills (TSSp) in adolescents aged 14 to 18 years in Spain. ESTUDES Surveys 2004–2014. * Linear time trend from 2004 to 2014 estimating prevalence of past 30 days, total consumption and nonmedical use of tranquilizers, sedatives, and sleeping pills, by sex, *p*- value

2.86% (5116) of the Spanish school population of both sexes reported nonmedical use of TSSp in the decade from 2004 to 2014. Consumption prevalence was greater among girls than among boys for all the study years (Fig. 1).

Tables 1 and 2 present prevalence data for nonmedical use according to sociodemographic variables, variables related to co-ingestion of other legal and illegal psychoactive drugs, risk perception, availability of these drugs, and the information received about drug abuse in schools. TSSp consumption increased with age for both sexes and significantly increased among female immigrant adolescents during the study years. Regarding the co-ingestion of other psychoactive substances, the prevalence of nonmedical use of TSSp was greater among students of both sexes who reported having consumed alcohol, tobacco and/or marijuana during the previous 30 days. Among those students who had consumed illegal psychoactive drugs other than marijuana during the last year, nonmedical use prevalence values were higher for girls. The results show that the percentage of female adolescents who perceived no problem with the nonmedical consumption of TSSp was superior to that of males. The prevalence of prior month nonmedical use of TSSp among those female adolescents who perceived TSSp as easy/very easy to obtain was 6.5%.

**Table 1:** Prevalence of nonmedical use of TSSp in Male adolescents, according to sociodemographic variables, use of licit and illicit psychoactive drugs and variables related with perceived health risk, perceived availability and School information. ESTUDES Survey

		2004	2006	2008	2010	2012	2014	Combined years	^a^Linear Trend *p*-value^a^
		N (%)	N (%)	N (%)	N (%)	N (%)	N (%)		
Age	14–15	64(1.18)	94(1.7)	118(1.84)	114(1.65)	84(1.79)	132(1.63)	606(1.64)	0.208
	16–18	170(2.29)	156(2.19)	233(2.74)	245(2.81)	274(2.99)	242(2.33)	1321(2.57)	0.287
Nationality	Immigrants	NA	240(2.04)	308(2.29)	313(2.24)	319(2.59)	316(1.92)	1495(2.2)	0.778
	Spanish	234(1.82)	10(1.18)	44(2.95)	46(2.9)	40(2.55)	58(2.89)	197(2.64)	0.204
Occupational status of parents	Unemployed both	16(9.83)	11(3.04)	33(5.4)	25(3.72)	38(4.7)	27(2.67)	150(4.14)	0.018
	Employed one	38(2.58)	37(2.32)	65(2.75)	68(2.37)	88(2.78)	84(2.17)	380(2.48)	0.689
	Employed both	179(1.6)	202(1.9)	254(2.12)	266(2.21)	232(2.35)	263(1.93)	1397(2.01)	0.043
Educational level of parents	No formal education	20(3.42)	8(1.93)	24(4.28)	22(5.1)	20(5.36)	12(3.05)	106(3.83)	0.388
	Primary school	89(1.9)	116(2.65)	101(1.96)	106(2.11)	108(2.33)	85(2.18)	605(2.18)	0.766
	Secondary school	28(1.66)	31(1.53)	53(2.23)	71(2.35)	69(2.47)	77(1.66)	330(1.98)	0.713
	Higher education	61(1.74)	74(2.1)	106(2.7)	125(2.65)	101(2.51)	122(1.87)	589(2.25)	0.871
Alcohol use in the past 30 days	No	54(1.14)	50(0.94)	82(1.3)	83(1.26)	35(0.88)	71(0.96)	374(1.09)	0.440
	Yes	180(2.21)	200(2.73)	269(3.12)	276(3.07)	324(3.26)	303(2.73)	1552(2.87)	0.049
Any cigarette smoking in the past 30 days	No	17(3.08)	126(1.33)	190(1.85)	194(1.61)	192(1.86)	213(1.45)	932(1.62)	0.754
	Yes	106(3.3)	124(3.96)	161(3.48)	165(4.65)	167(4.66)	161(4.2)	885(4.03)	0.038
Any marijuana use in the past 30 days	No	112(1.2)	122(1.25)	166(1.44)	177(1.4)	224(1.98)	214(1.37)	1014(1.45)	0.035
	Yes	122(3.43)	127(4.54)	186(5.41)	183(6.05)	135(5.26)	160(5.46)	913(4.99)	0.001
Any illicit psychoactive drug use other than marijuana in the last 12 months	No	151(1.27)	155(1.3)	245(1.72)	232(1.55)	253(1.9)	280(1.55)	1316(1.56)	0.020
	Yes	83(8.83)	95(13.92)	106(15.19)	127(20.19)	106(18.76)	94(20.2)	610(15.35)	0.000
Perceived health risk for consumption of TSSp	No/few problems	67(6.02)	85(5.97)	126(7.10)	107(6.12)	94(8.48)	79(4.85)	557(6.34)	0.699
	Quite a few/many problems	120(1.29)	130(1.48)	143(1.47)	164(1.81)	184(2.12)	204(1.66)	946(1.64)	0.009
Perceived availability of TSSp	Impossible/very difficult to obtain	29(0.87)	32(0.9)	55(1.26)	49(1.44)	62(1.66)	86(1.55)	313(1.31)	0.004
	Easy/very easy to obtain	175(2.7)	175(3.46)	229(4.26)	222(3.97)	196(4.89)	196(3.92)	1193(3.79)	0.000
Information on drugs received at school	No	65(1.97)	95(1.99)	102(2.78)	111(3.04)	111(3.47)	101(2.48)	584(2.58)	0.012
	Yes	169(1.77)	155(1.97)	213(2.01)	226(1.98)	225(2.21)	242(1.76)	1230(1.94)	0.848

*NA* not available

^a^Linear time trend from 2004 to 2014 estimating prevalence of past 30 days nonmedical use of tranquilizer, sedatives and sleeping pills by demographic subgroup

**Table 2:** Prevalence of nonmedical use of TSSp in Female adolescents, according to sociodemographic variables, use of licit and illicit psychoactive drugs and variables related with perceived health risk, perceived availability and School information. ESTUDES Survey

		2004	2006	2008	2010	2012	2014	Combined years	^a^Linear Trend *p*-value^a^
		N (%)	N (%)	N (%)	N (%)	N (%)	N (%)		
Age	14–15	138(2.56)	153(2.45)	200(2.94)	233(3.15)	161(3.4)	272(3.13)	1157(2.95)	0.013
	16–18	242(3.31)	241(3.16)	311(3.68)	360(4.01)	416(4.68)	464(4.51)	2032(3.95)	0.000
Nationality	Immigrants	NA	360(2.82)	445(3.28)	511(3.52)	487(4.04)	638(3.81)	2441(3.51)	0.000
	Spanish	380(3.00)	34(3.10)	65(3.96)	81(4.45)	89(5.68)	98(4.4)	368(4.39)	0.084
Occupational status of parents	Unemployed both	2(1.36)	16(3.76)	24(5.15)	39(5.38)	50(6.13)	72(6.64)	203(5.5)	0.007
	Employed one	67(3.99)	61(3.29)	104(3.57)	150(4.40)	156(4.29)	178(4.09)	715(4.01)	0.292
	Employed both	310(2.87)	317(2.74)	383(3.23)	404(3.30)	371(4.04)	486(3.59)	2271(3.28)	0.000
Educational level of parents	No formal education	16(2.97)	17(2.55)	22(3.26)	16(2.81)	22(4.76)	18(3.19)	111(3.19)	0.374
	Primary school	129(2.62)	158(2.97)	220(3.69)	214(3.67)	198(3.99)	194(4.03)	1113(3.5)	0.000
	Secondary school	49(2.9)	64(2.88)	64(2.82)	124(3.9)	119(4.31)	180(3.81)	600(3.56)	0.015
	Higher education	127(3.76)	97(2.91)	127(3.46)	171(3.88)	172(4.48)	246(3.9)	941(3.77)	0.097
Alcohol use in the past 30 days	No	81(1.79)	132(2.27)	156(2.52)	176(2.54)	78(2.21)	139(2.01)	763(2.25)	0.888
	Yes	299(3.66)	262(3.26)	354(3.91)	417(4.4)	498(4.94)	597(4.95)	2427(4.27)	0.000
Any cigarette smoking in the past 30 days	No	15(2.71)	214(2.23)	262(2.59)	346(2.98)	310(3.22)	413(2.84)	1560(2.78)	0.014
	Yes	188(4.57)	180(4.24)	248(4.83)	247(5.19)	266(6.68)	323(7.25)	1452(5.44)	0.000
Any marijuana use in the past 30 days	No	231(2.34)	291(2.56)	362(2.87)	417(3.00)	417(3.52)	530(3.16)	2246(2.94)	0.000
	Yes	149(5.3)	103(4.12)	149(5.66)	176(7.08)	160(8.97)	206(9.44)	943(6.55)	0.000
Any illicit psychoactive drug use other than marijuana in the last 12 months	No	318(2.61)	360(2.67)	456(3.06)	524(3.26)	512(3.82)	683(3.64)	2855(3.21)	0.000
	Yes	61(12.52)	34(8.86)	54(16.13)	68(23.76)	64(29.05)	53(24.62)	334(17.35)	0.000
Perceived health risk for consumption of TSSp	No/few problems	101(8.37)	106(7.70)	140(9.4)	148(9.05)	146(14.33)	183(10.99)	825(9.82)	0.001
	Quite a few/many problems	249(2.47)	236(2.20)	285(2.51)	338(3.09)	339(3.60)	417(3.1)	1864(2.82)	0.000
Perceived availability of TSSp	Impossible/very difficult to obtain	23(0.71)	64(1.59)	61(1.34)	65(1.96)	75(2.17)	101(1.94)	389(1.63)	0.000
	Easy/very easy to obtain	320(4.76)	278(4.8)	361(6.24)	432(6.79)	388(9.21)	448(8.30)	2226(6.50)	0.000
Information on drugs received at school	No	98(3.42)	154(3.14)	121(3.29)	154(4.54)	159(4.87)	176(4.22)	861(3.87)	0.003
	Yes	282(2.87)	240(2.68)	369(3.3)	423(3.35)	402(3.99)	533(3.74)	2248(3.36)	0.000

*NA* not available

^a^Linear time trend from 2004 to 2014 estimating prevalence of past 30 days nonmedical use of tranquilizer. Sedatives and sleeping pills by demographic subgroup

Table 3 shows the results of two multivariate analysis models performed using logistic regression- one for males and the other for females. They show the independent effect of each study variable, adjusted for the other variables, on TSSp nonmedical use in our sample.

**Table 3:** Multivariable logistic regression of past 30 days nonmedical use of TSSp among the school population aged 14 to 18 years in Spain. ESTUDES Survey 2004–2014

		Male		Female	
		OR (95% CI)	aOR (95% CI)	OR (95% CI)	aOR(95% CI)
Occupational status of parents	Employed both	1	1	1	1
	Employed one	1.23(1.031.48)	1.09(0.87–1.38)	1.23(1.10–1.37)	1.17(1.01–1.35)
	Unemployed both	2.10(1.70–2.60)	1.59(1.13–2.24)	1.71(1.40–2.11)	1.37(1.04–1.80)
Alcohol use in the past 30 days s	No	1	1	1	1
	Yes	2.67(2.31–3.09)	1.57(1.26–1.95)	1.94(1.74–2.16)	1.47(1.25–1.73)
Any cigarette smoking in the past 30 days	No	1	1	1	1
	Yes	2.54(2.23–2.90)	1.21(0.98–1.49)	2.00(1.83–2.21)	1.43(1.24–1.67)
Any marijuana use in the past 30 days	No	1	1	1	1
	Yes	3.57(3.14–4.07)	1.54(1.24–1.91)	2.31(2.09–2.55)	1.26(1.07–1.48)
Any illicit psychoactive drug use other than marijuana in the last 12 months	No	1	1	1	1
	Yes	11.44(9.88–13.25)	6.21(4.97–7.77)	6.32(5.43–7.35)	3.38(2.70–4.23)
Perceived health risk for consumption of TSSp	Quite a few/many problems	1	1	1	1
	No/few problems	4.07(3.55–4.67)	3.04(2.55–3.62)	3.74(3.36–4.17)	2.92(2.55–3.33)
Perceived availability of TSSp	Impossible/very difficult to obtain	1	1	1	1
	Easy/very easy to obtain	2.97(2.50–3.53)	2.20(1.77–2.74)	4.18(3.64–4.82)	3.51(2.98–4.14)

*OR* odds ratio, *aOR* adjusted OR, *CI* confidence interval

When analyzing patterns of nonmedical use among female adolescents, the variables related to consumption of psychoactive substances in the previous 30 days, which were independently and significantly associated with a greater probability of TSSp nonmedical use were alcohol, tobacco and marijuana consumption (aOR = 1.26, 95% CI 1.07–1.48). Female adolescents who reported having consumed some type of illegal psychoactive drug, other than marijuana, in the previous 12 months, were 3.38 times more likely to make nonmedical use of TSSp (aOR = 3.38, 95% CI 2.70–4.23).

Among these adolescents, the perceived risks in consuming these substances and their availability (aOR = 3.51, 95% CI 2.98–4.14) acted as predicting factors for TSSp nonmedical use.

Among the male school adolescents in our study, alcohol and marijuana were also significantly associated with TSSp nonmedical use. Consumption in the previous year of any type of illegal psychoactive substance, other than marijuana, was the variable showing the greatest value in male teenagers, with a statistically significant association, presenting aOR values of 6.21 (95% CI 4.97–7.77). The perceived risk for the use of these substances (aOR = 3.04, 95% CI 2.55–3.62) and their availability showed a statistically significant association in these male teenage students.

When analyzing trends for TSSp nonmedical use from 2004 to 2014, female teenage students presented an OR = 1.04 (95% CI 1.02–1.05), taking 2004 as a reference. Confounding variables were controlled for so that statistical signification was maintained (OR: 1.06, 95% CI 1.04–1.08), which means that the nonmedical use of TSSp among adolescent females of the Spanish school population varied about 6% every year during the analyzed period of time.

Among male Spanish adolescent students, no significant association was found when analyzing TSSp nonmedical use trends during the study decade.

## Discussion

The addition of the gender perspective to the study of drug addictions in recent years has generated new ways of understanding the usage patterns of new consumers [10, 15, 26, 27]. This approach allows for the identification of male and female consumption patterns and makes female usage more visible.

When analyzing TSSp nonmedical use among Spanish adolescents, our results indicate that for the study decade, comprised from 2004 to 2014, the prevalence of nonmedical use of prescription medicine has experienced a significant increase of around 6 % in the female school population residing in Spain (aOR = 1.06, 95% CI 1.04–1.08). These results agree with those obtained during 2002 and 2005 by the United States (US) National Survey on Drug Use and Health (NSDUH), which indicated that US female adolescents presented higher rates of nonmedical use of tranquilizers and sedatives than male adolescents [28]. The investigation performed by Young et al. by means of the systematic review of the scientific bibliography on nonmedical use of prescribed medicines in adolescents found that, in most of the studies, female gender was more associated with tranquilizer use [29]. Also, the Brazilian study by Opaleye et al. shows that female adolescents have twice the probability (aOR = 2.19; 95% CI: 1.75–2.75) of reporting nonmedical use of these medicines than males [20].

When identifying patterns for TSSp nonmedical use among the Spanish school population, we found that the only socio-demographic variable in our study that acted as a predictor for the nonmedical use of these drugs, both in male and female adolescents, was unemployment for both parents. Some studies have indicated that students with a low socio-economic status present a greater tendency to consume substances in their adolescence [30, 31]. This relationship is described in studies such as that by Bali et al., in which they noted that individuals with family incomes lower than $20,000 were more likely to make nonmedical use of prescription drugs [32]. Also, in the Canadian study performed with data from the Canadian Health Behavior in School-Aged Children study, results indicated that students with a lower socioeconomic status (SES) were 2.41 times more likely to make recreational use of any type of drug [24].

When we analyzed the consumption of legal psychoactive substances and their relationships with the nonmedical use of TSSp, we found that alcohol use and smoking in adolescent females were significantly associated with the nonmedical use of these prescription drugs. This revealed a growing trend towards the incorporation of female adolescents into the consumption ranks of these legal drugs, surpassing male consumption rates [23, 26]. Recent research shows that the gender gap in alcohol consumption is decreasing [33]. These changes in normative standards, mainly for alcohol consumption, are contributing to a series of gender gaps within Spanish society that could be related to the fact that female adolescents associate alcohol consumption with pleasure, and they now consume alcohol in public places [34].

With regard to adolescents, cannabis is the only illegal psychoactive substance whose consumption has increased among the Spanish female population in recent years, probably due to the normalization and acceptance of such use, as well as its low risk perception [35]. In our study, marijuana consumption was associated with TSSp nonmedical use in the school population of both genders. Several studies indicate that the probability of using marijuana increases among those adolescents who report nonmedical use of benzodiazepines and anxiolytics [36]. A recent US study assessing longitudinal associations between the nonmedical use of sedatives and anxiolytics during adolescence found that approximately 92.9% of the adolescents also consumed other psychoactive substances, mainly alcohol and cannabis [37].

In line with that, illegal psychoactive substance consumption is a risk factor related to starting nonmedical use of prescription drugs among adolescents. A cross-national study of the nonmedical use of prescription medications in five European countries found that 28% of those who engaged in nonmedical use of sedatives in the previous year had also consumed illegal drugs [5]. Our study showed that the use of illegal psychoactive substances was the factor most strongly associated with TSSp nonmedical use among school adolescents of both genders. Male Spanish adolescents who reported that they consumed illegal drugs were six times more likely to make nonmedical use of these medicines. Similar results were obtained in a study performed in 31 European countries with data from the European School Survey on Alcohol and Other Drugs, the results of which showed that those adolescents who engaged in nonmedical use of tranquilizers and sedatives were 3.48 times more likely to consume illegal drugs [23]. In recent years, there has been an increase in illegal drug consumption among younger females although differences between female and male consumption rates remain considerable. These data have given rise to the so-called “convergence hypothesis”, which states that differences between males and females in drug use are lessening and are expected to continue in this same vein [38].

The use of drugs among adolescents has increased due to the low risk perception associated with their consumption. Therefore, it will be necessary to identify the factors that have led to this perception and to the corresponding increase in addictive drug behavior rates among adolescents [39]. The low risks perceived for TSSp nonmedical use among Spanish teenage students makes them 3 times more likely to consume drugs than those adolescents who perceive such consumption as highly risky. This agrees with data from a national survey performed with adolescents of both sexes, the results of which showed that those participants with a low risk perception of consumption (aOR = 1.53 95% CI 1.09–2.15) were the most prone to nonmedical use of tranquilizers and sedatives [21]. If we take together the low risk perception and the ease of obtaining TSSp for our adolescents, mainly among girls (6.50% indicate that they find it easy or very easy to obtain them), the problem is aggravated, since these two variables are associated with the nonmedical use of these medicines. We should bear in mind that drugs are often used by other family members through medical prescription and are, therefore, available inside adolescents’ homes. This can make adolescents conclude that their behavior is not that at odds with social standards, since their use complies with adult models. The access availability of TSSp is also reflected in other studies which show that 20.7% of students agreed that obtaining tranquilizers and sedatives was “very easy” or “quite easy” and indicated that the first drug they ever abused belonged to their parents [33, 40].

The scientific community has accepted the incorporation of the gender perspective to drug consumption, since women and men respond to different constraints and, therefore, any analysis, strategy or action, should separately contemplate and study the aspects and factors conditioning them. The question analyzes real life and circumstances for males and females in a separate manner with the objective of identifying how gender constraints affect the issue of drug consumption.

One of the main strengths of this study is the application of a gender perspective to a nationally representative sample in order to study the prevalence of the nonmedical use of TSSp in school populations over a decade. Given that the methodology of the ESTUDES survey is similar to that used in other European Union countries and the United States, we feel empowered to make international comparisons.

However, our study is subject to a series of limitations. The first limitation of surveys about drug abuse comes from the cross-sectional nature of the study data, which does not allow for establishing the direction of the associations discovered.

The second limitation comes from using self-declared data. Thus, the prevalences obtained to generate nonmedical use profiles for tranquilizers, sedatives and/or sleeping pills could be underestimated. This is because, due to sociocultural judgments surrounding the consumption of such substances, some school adolescents will have reservations about openly confessing their prescribed or self-medication consumption of these medications.

We also need to point out that the non-response rate (between 9 and 17.1% (ESTUDES)), affects our estimations of reported consumption, since those adolescents who refused to participate could share certain characteristics related to consumption, although the direction of the effect cannot be determined [35].

## Conclusions

This study shows that trends in tranquilizer, sedative and/or sleeping pill nonmedical use among female adolescents in the school population residing in Spain increased by 6% in the decade comprised from 2004 to 2014.

When analyzing TSSp, nonmedical use patterns for alcohol, tobacco and marijuana act as prediction variables for adolescent females. Special mention should be made of adolescents from both sexes, but mainly among male adolescents, who reported that they consumed illegal psychoactive substances other than marijuana, because there is a higher probability of misusing these drugs.

Finally, a low perception of consumption risk and the ease in obtaining these drugs also presented a strong relationship with tranquilizer, sedative and sleeping pill nonmedical use among Spanish adolescent students of both sexes.

Addressing the consumption of drugs from a gender perspective involves taking into account the differences and particularities that gender introduces into use patterns for these substances and motivation for using drugs, as well as the effects and consequences derived from their use. We believe that this study provides interesting and valuable results from the point of view of preventing the consumption of psychoactive substances, such as TSSp, among adolescents, and may effectively contribute to the development of prevention programs for drug consumption in adolescence, given the significant increase that has taken place in just one decade.

## Data Availability

The datasets generated and/or analyzed during the current study are available in the Spanish National Drug Plan repository, http://www.pnsd.mscbs.gob.es/en/profesionales/sistemasInformacion/sistemaInformacion/encuestas_ESTUDES.htm

## Abbreviations

95%CI: 95% confidence interval
aOR: Adjusted Odds Ratio
OR: Crude Odds Ratio
ESPAD: European School Survey Project on Alcohol and other Drugs
ESTUDES: Spanish State Survey on Drug Use in Secondary Education
NA: Not available
NSDUH: National Survey on Drug Use and Health
TSSp: Tranquilizers, sedatives, and sleeping pills
US: United States
